# Exploring the Limitations of Event-Related Potential Measures in Moving Subjects: Pilot Studies of Four Different Technical Modifications in Ergometer Rowing

**DOI:** 10.3390/s20195618

**Published:** 2020-10-01

**Authors:** Holger Hill

**Affiliations:** Mental mHealth Lab, Institute of Sports and Sports Science, Karlsruhe Institute of Technology, 76131 Karlsruhe, Germany; holger.hill@kit.edu

**Keywords:** event-related potentials, ERP, EEG, movement artefact, rowing movement

## Abstract

Measuring brain activity in moving subjects is of great importance for investigating human behavior in ecological settings. For this purpose, EEG measures are applicable; however, technical modifications are required to reduce the typical massive movement artefacts. Four different approaches to measure EEG/ERPs during rowing were tested: (i) a purpose-built head-mounted preamplifier, (ii) a laboratory system with active electrodes, and a wireless headset combined with (iii) passive or (iv) active electrodes. A standard visual oddball task revealed very similar (within subjects) visual evoked potentials for rowing and rest (without movement). The small intraindividual differences between rowing and rest, in comparison to the typically larger interindividual differences in the ERP waveforms, revealed that ERPs can be measured reliably even in an athletic movement such as rowing. On the other hand, the expected modulation of the motor-related activity by force output was largely affected by movement artefacts. Therefore, for a successful application of ERP measures in movement research, further developments to differentiate between movement-related neuronal activity and movement-related artefacts are required. However, activities with small magnitudes related to motor learning and motor control may be difficult to detect because they are superimposed by the very large motor potential, which increases with force output.

## 1. Introduction

The investigation of brain functions with noninvasive methods such as functional magnetic resonance imaging (fMRI), magnetencephalography (MEG), electroencephalography (EEG), near-infrared spectroscopy (NIRS), and transcranial magnetic stimulation (TMS) is typically limited to laboratory settings. This is because the systems are very large and cannot be moved, and head movements must be avoided (fMRI, MEG), or head and body movements generate large movement artefacts. Within the last decade, there has been a rapidly increasing interest in investigating brain functions in ecological settings (e.g., cognitive or neuropsychological processes in interaction with a natural environment or in a social context; and movement analysis and motor learning), which require portable systems [1,2,3,4,5,6]. Especially in the field of movement research/motor learning, laboratory settings are strongly limiting, because only simple finger or hand or arm movements can be investigated, and it is questioned whether the results of these studies can be transferred to complex movements [7,8,9]. In principle, NIRS and EEG are suited for measures in moving subjects, because the sensors are small and fixed to the head (rather than the head being fixed to the sensor), and the necessary electronics and recording devices can be built small enough. NIRS, similar to fMRI, measures the hemodynamic response related to specific brain processes with a low temporal resolution. Spatially, it is restricted to cortical layers close to the skull. Ref. [10] used a modified NIRS system successfully in a cognitive task when subjects were walking around. Ref. [11] developed a miniaturised wearable functional NIRS system and tested it when subjects performed a left hand gripping task (i) sitting still on a bicycle, (ii) pedaling indoor on a stationary training bicycle, and (iii) during outdoor bicycle riding. The event-related data showed that the task was performed successfully and comparably in all three conditions, whereas data loss was highest in real cycling (about 35%) but much lower in indoor cycling (7.5%) and under rest (5%).

**Figure 1:**
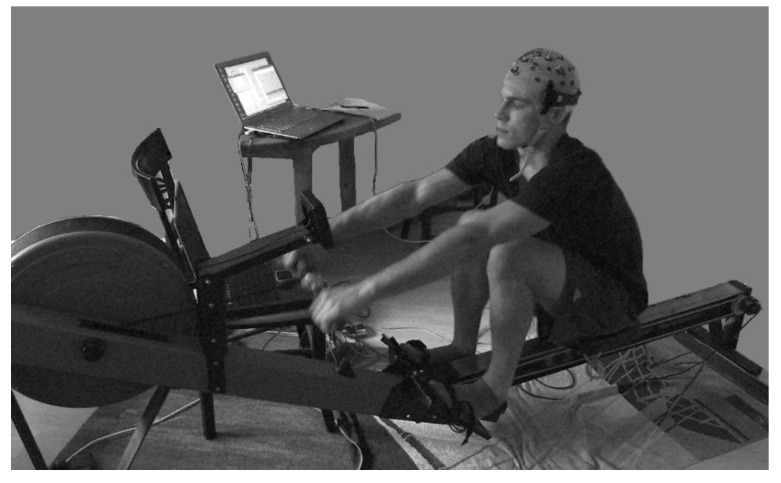
Electroencephalography (EEG) recording during ergometer rowing in pilot Study 3. The Concept II (Model C) indoor rower was used for the pilot studies. The Concept II models are the most frequently used ergometers for training in competitive rowing and performance diagnostics. In the drive phase of the rowing cycle, the rower’s pull accelerates an air resistance braked flywheel in the round cage. In the recovery phase, the rower moves in the opposite direction on the sliding seat, preparing for the next pull. A monitor displays the stroke rate, time, distance rowed, power per stroke, mean power, and calories burned. On the chair at the right side in front of the rower is the laptop for stimulus presentation. The laptop on the table recorded the behavioral and EEG data (photo use with participant’s permission).

Event-related potential (ERP) measures, which are the focus of this paper, have the well-known limited spatial resolution but a high temporal resolution, which is essential to analyse complex movements: for example, the time course of feedback and feedforward processing in visuomotor learning [12,13]. Conventional EEG systems are very sensitive to mechanical (cable and electrode movements) and physiological (electromyogram (EMG) of head and neck muscles, and sweating) movement artefacts [14]. If cognitive processes in a moving subject rather than the movement itself are the focus of interest, data preprocessing algorithms informed by the behavioural movement data can be used to clean the EEG data from movement-related (neuronal and artefactual) activity [15,16]. If the motor-related activity is of interest, advanced data preprocessing algorithms such as independent component analysis (ICA) [17] can be used to correct such artefacts. However, a study measuring ERPs during walking and running on a treadmill showed that data loss was very high (on average 130 of 248 EEG channel signals remained), even using a system with active electrodes that are considerably less prone to artefacts than conventional passive electrodes [18,19]. Furthermore, the usability of conventional EEG systems such as the one used in these studies is limited for measures with moving subjects. In movement tasks with only marginal head movements, such as cycling on an ergometer, these laboratory systems combined with ICA-based artefact correction can be applied successfully for EEG measures [20]. Ref. [21] found in a high-intensive cycling exercise an increase in spectral power when the athletes were fatigued. If spectral changes of higher EEG frequencies (alpha to gamma) are the focus of interest, such as in ref. [21], artefacts directly coupled to movement execution are outside of this frequency range, because movement frequencies are considerably lower. However, the harmonics of these movement frequencies may occur, which have to be considered. With fully moving subjects, in contrast, artefacts are more difficult to handle. In a cocktail party study (including eating, drinking, chatting, etc.) with ten subjects wearing self-made (noncommercial) wireless EEG headsets, about 40% of the data were lost due to artefacts in contrast to 4% in two laboratory studies [22]. Despite this high data loss, these studies revealed valuable ERP [18,19] or spectral EEG [22] results. However, especially in movement research, the method of choice is to avoid the generation of mechanical artefacts beforehand by technical modifications. This approach was used in the four pilot studies reported in this paper that tested the suitability of different technical solutions to measure ERPs during ergometer rowing (Figure 1). Especially for analysing motor-related brain activity with ERPs, rowing is well suited. It is a cyclic movement with a high number of repetitions, and the degrees of freedom of the movement are limited by the biomechanical constraints of the equipment (boat and scull/oar, or ergometer). Furthermore, in contrast to e.g., cycling or kayaking, the rowing movement is composed of different (more or less) distinct movement elements, and the movement frequency is lower (20–40 strokes/min vs. 60–120 revolutions/min in cycling). Finally, the biomechanical data (dynamics and kinematics of the movement, and boat and scull/oar movement), which are partly necessary for ERP analysis, can be measured meanwhile with relative ease.

The most critical question before pilot Study 1 (see below) was whether movement artefacts distort the EEG data completely or if there are systematic artefacts that can be controlled and either (partly) excluded or corrected in offline analysis. To test this question, a reliability check was made. A standard visual oddball task was applied in a rest condition (without movement) and during ergometer rowing. Similar—not movement-related—ERP activities during rest and during rowing would show that ERPs can be measured reliably in a moving subject. The second methodological question—concerning the analysis of motor behavior with ERPs—is: Can movement-related artefacts be identified and separated from motor-related neuronal activity? As an indicator for a reliable measure of motor-related activity, at least a motor potential (MP), a negative activity related to force output, should be expected. Ref. [23] showed in a study that used isometric elbow flexions that the amplitude of the MP (labeled motor-related cortical potential (MRCP) in this study) correlates very high with EMG activity (r > 0.8) and the generated muscle force (r = 0.95). Furthermore, in an fMRI study, a high correlation between isometric force (using a hand-grip dynamometer) and activity in the primary motor cortex was found [24]. The MP/MRCP must be generated by different generators because it can already be evoked when a muscle activation is only imagined. This should originate in the supplementary motor area (SMA) [25]. A second part of the MP is thought to be related to the control of muscle activation by the primary motor cortex.

The purpose of this series of four consecutive pilot studies was as follows: first, to test if ERPs can in principle be measured during an athletic movement pattern such as rowing; second, to explore the limitations due to movement artefacts; and third, to test if movement artefacts can be reduced. Methodologically, the impact of physical and physiological artefact sources was investigated by comparing ERP waveforms measured at rest and during movement. For the existing data material, this physical approach is the method of choice compared to a statistical analysis of parametrised abstract data derived from the original data. Study outlines: In pilot Study 1 (conducted in 2005), a small, purpose-built 20-channel system was used with a preamplifier connected to the head and electrodes with shortened and fixed cables mounted to a standard electrode cap. Since this approach was only partly successful, the second approach (Study 2) was performed. This experiment involved the use of a system that was available in our lab at that time (2008) with active electrodes with built-in preamplifiers, and the amplifier was worn in a backpack. In pilot Study 3 (2013), as the newest improvement in EEG technology, a small head-mounted EEG system with wireless data transmission was combined with the electrodes and cap used in Study 1. In Study 4 (2018), finally, the headset used in Study 3 was combined with active electrodes, and movement intensity (force and speed) was systematically varied to investigate the influence of these factors on data quality.

## 2. Pilot Study 1

### 2.1. Materials and Methods

A preliminary test using a standard EEG system (NeuroScan SynAmps with passive electrodes) by wearing the 32-channel preamplifier headbox in a backpack revealed no satisfying results. Therefore, to reduce the generation of artefacts due to cable and electrode movements, a small purpose-built (according to ref. [26]) battery-powered 20-channel system (based on positive previous experiences with a similar three-channel system) was used with an occipitally mounted preamplifier (differential amplifier, gain = 30; hardware filters: 0.27 Hz passive RC highpass; 30 Hz 2nd-order Bessel lowpass). After a second amplifier stage (total gain = 3600), the signals were digitised using a BEST system (Dr. Grossegger & Drbal Company, Korneuburg, Austria; sample rate 256 Hz, resolution 0.3 μV/bit). An electrode cap (EasyCap, EasyCap GmbH, Herrsching-Breitbrunn, Germany, www.easycap.de) and 22 gold electrodes (Grass Instrument Company, Quincy, MA, USA) with shortened cables were used. Electrode caps with electrodes fixed to the cap have the disadvantage that the contact of some electrodes with the skin can be poor, depending on head shape (that is, if there are dents in the skull, the distance between the cap and skin can be too large). Therefore, the standard adaptors inserted in the cap were removed, the adaptor holes were enlarged, and the electrodes were fixed using a highly viscous conductive paste (Elefix, Nihon-Kohden Europe GmbH, Rosbach, Germany). EEG was recorded from 20 sites (midline: FPz, Fz, FCz, CPz, Pz, and Iz; Left/right: FC1/2, FC3/4, FC5/6, C3/4, CP1/2, CP3/4, left and right mastoid) covering mainly the sensorimotor area and Iz to assess visual evoked potentials (VEPs). Data were recorded using Cz as reference and rereferenced offline (see results for details). 

Rowing force was measured with a modified ergometer handle using strain gauges (according to the measure and analysis of oar forces in rowing [27]), and movement of the sliding seat was measured with a potentiometer. Biomechanical data were recorded with a second computer, synchronised using triggers of the Presentation software, and added to the EEG data file offline. 

A visual oddball task was used with 180 black–white fullscreen checkerboards (frequent), 60 red–white checkerboards (deviant), and 60 gray crosses (target), which had to be counted. The stimulus duration was 100 ms, and the interstimulus interval varied randomly between 800 and 1000 ms. The oddball task is a standard paradigm and generates robust ERP components: VEPs at occipital sites that are generated in the visual cortex when visual stimuli are presented. The P300, which is most prominent at centroparietal sites (around CPz, Pz), is generated when attention is shifted to a target stimulus. Stimuli were presented asynchronous to the temporal pattern of the rowing movement on a computer screen (screen refresh rate 60 Hz) besides the ergometer using the Presentation software (Neurobehavioral Systems, www.neurobs.com), allowing monitoring the display without head movements, although eye movement artefacts may be generated. Alternatively, an acoustical stimulation could be used. However, because the auditory cortex is closer to the sensorimotor cortex and to the mastoids than the visual cortex, this will probably lead to an extended signal overlay of motor-related and auditory-evoked potentials. 

The EEG was analysed using the Vision Analyzer 1.0 software (BrainProducts, Gilching, Germany). EEG data were digitally filtered with a 16 Hz/24 dB Butterworth zero phase lowpass, which was segmented into epochs of −200 to 800 ms around stimulus onset (oddball task) and baseline corrected (−200 to 0 ms). After applying a semiautomatic procedure for artefact detection (amplitude criterion ±100 µV, gradient 25 µV/sample), the complete datasets were inspected visually for further artefacts, surviving the automatic rejection. Segments with blink or eye movement artefacts (detectable at frontal sites) were excluded completely. Traces of single channels containing other clearly visible large artefacts were removed. If there were more than five contaminated traces, the whole segment was removed. ERPs were computed for each stimulus category of the oddball task. Motor-related ERPs were computed triggered by the force onset at the beginning of the rowing stroke. 

As a measure of signal quality, the signal-to-noise ratio (SNR) was computed (approximately the variance of the ERP waveform divided by the variance of the EEG segments underlying the average, see Appendix A for details). Furthermore, the correlation coefficients (Pearson) between the ERP waveforms measured during rowing and rest were computed (adopted from ref. [28]). The r-value would not change if the waveform is attenuated in one condition. However, if the waveform is distorted by artefacts, the r-value will decrease. Should an attenuation effect be considered as well, Lin’s concordance correlation coefficient [29] could be used. Alternatively, the area of the difference between the two waveforms could be computed and related to the areas of the original waveforms. However, this procedure, which was developed to measure within-crew coordination in rowing [27], is not common. 

Before performing this pilot study, a statement of the ethics committee of the German Society of Psychology was obtained for a grant proposal. This stated no ethical concerns about this type of investigations. Written informed consent was obtained (respectively from a parent when under 18). Three male subjects (aged 47, 13, and 10 years, who are referred to here as H, J, and M, respectively) performed (i) the oddball task sitting still on the ergometer, (ii) rowing without stimulation, and (iii) the oddball task during rowing. Subjects were instructed to row in a recreational mode (e.g., 130 W for subject H) during the oddball task and to keep rowing power and stroke rate (20/min) constant, using the performance monitor of the ergometer for control. The rest condition of the oddball task was always performed first to avoid sweating artefacts.

### 2.2. Results

**Figure 2:**
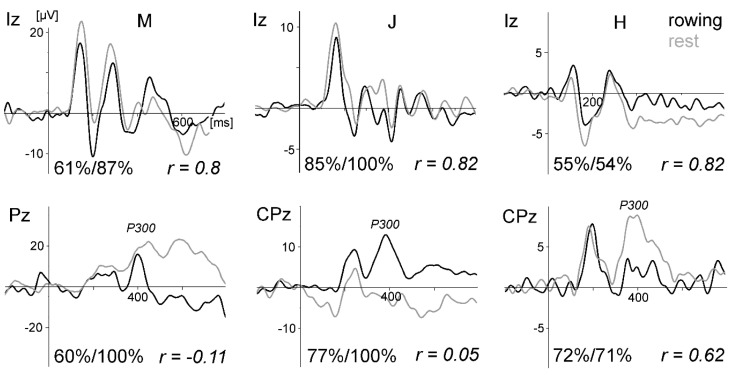
Event-related potential (ERPs) of the visual oddball task comparing rowing and rest for all three subjects (M, J, and H) of pilot Study 1. Upper graphs: visual evoked potentials (VEPs) evoked by the frequent checkerboard stimulus at electrode site Iz (referenced to Cz to obtain a larger and cleaner signal because VEP activity and electromyogram (EMG) artefacts are lower at Cz compared to the mastoids). Below: the P300 waveform at Pz/CPz (linked mastoid reference for M and H, and right mastoid for J due to the lost left mastoid channel). The vertical line marks the stimulus onset. The numbers at the bottom of each graph present the percentage of artefact-free trials included in the averages for rowing and rest of the chosen electrode site and the correlation coefficients (Pearson) of the two waveforms. The high dropout rate for subject H was mainly due to eye-blink artefacts. For subject M, three channels (FCz, CPz, and CP4) were lost completely due to artefacts during rowing: for subject J, the left mastoid channel was lost. Furthermore, a P300 is missing during rest for J, because he did not count the target because of an imprecisely given instruction. For M and H, the P300 was larger during rest. The ERPs of the deviant stimulus condition are not displayed, because they showed results similar to those of the frequent condition.

Figure 2 displays ERPs of all three subjects for the oddball task. Especially the VEPs were very similar between rowing and rest, and intraindividual differences were much smaller than the interindividual differences. This result demonstrates that standard ERPs not time-locked to the rowing movement can be measured during rowing, despite the fact that subjects M and J had no or only marginal rowing experience. The SNR revealed for the VEP waveform at Iz always has higher values for the rest condition than for rowing (mean 0.207 vs. 0.038). T-test: t (2) = 16.2, *p* = 0.0038.

In contrast to the VEPs, the motor-related ERPs were very noisy and showed large artefacts with large inter- and intraindividual (implemented by varying force output and stroke rate) differences and were therefore not interpretable. One observable source of large artefacts was due to the movements of the cable connecting the head-mounted preamplifier with the second amplifier unit, which led to movements of the preamplifier and electrode cables.

## 3. Pilot Study 2

### 3.1. Materials and Methods

The second approach used a system with active electrodes at the Department of Psychology, University of Frankfurt. This system suppresses artefacts due to cable movements; however, electrode movements cannot be avoided completely. During rowing and rest, the amplifiers were worn in a backpack and connected via fibre-optic cables to a PC. EEG was recorded continuously with BrainAmp DC amplifiers (BrainProducts, Gilching, Germany; sample rate 250 Hz, resolution 0.1 µV/bit, input impedance 10 MOhm) using an equidistant EasyCap (EasyCap GmbH, Herrsching-Breitbrunn, Germany, www.easycap.de) with 62 sintered Ag/AgCl electrodes and built-in preamplifiers (BrainProducts ActiCap System). Eye blinks and movements were monitored with supra- and infra-orbital electrodes and with electrodes on the external canthi. The vertex electrode was used as the reference. To avoid injuries due to skin abrasion, electrode impedances were kept at 20 kOhm, which is more than sufficient from electrical engineering principles [30,31]. The EEG was analysed such as in Study 1 with slightly different filter settings (0.5 to 20 Hz bandpass). The averages were rereferenced (average reference transformation [32]), and the reconstructed vertex reference was added to the data, resulting in 61 EEG channels. The ergometer and its measuring equipment and the experimental procedure were the same as in Study 1. The task was performed by two female students (F and K, aged 24 and 26) without rowing experience and an experienced male rower (H, aged 50). Written informed consent was obtained. Subjects rowed in a recreational mode during the oddball task with a stroke rate of 20/min.

### 3.2. Results

**Figure 3:**
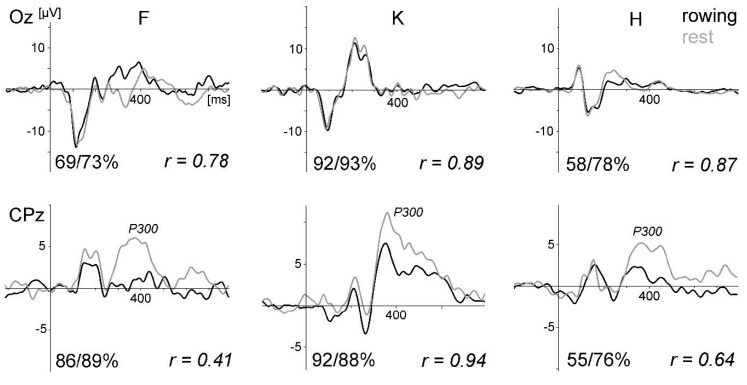
ERPs of the visual oddball task comparing rowing and rest for all three subjects (F, K, and H) of pilot Study 2. Upper graphs: VEPs evoked by the frequent checkerboard stimulus at electrode site Oz (average referenced). Below: the P300 waveform at CPz, which was larger during rest. The vertical line marks the stimulus onset. The numbers at the bottom of each graph present the percentage of artefact-free trials included in the averages for rowing and rest of the chosen electrode site and the correlation coefficients. The high dropout rate for subject H was due to eye-blink/eye-movement artefacts. For subject F, the data loss at occipital sites was due to neck muscle activity. Deviant stimulus condition is not shown.

Figure 3 displays the ERPs of all three subjects for the oddball task. As in Study 1, the VEPs were very similar between rowing and rest, the P300 was smaller during rowing, and the intraindividual differences were much smaller than the interindividual differences. The SNR values were always higher for the rest condition (VEP mean at Oz: 0.315 vs. 0.135, t (2) = 11.7, *p* = 0.0072). 

Motor-related activity, on the other hand, was not dominated by the expected motor potential in all conditions. To reject artefacts, the Infomax ICA algorithm implemented in the Vision Analyzer 1.0 software was applied. Components containing artefactual elements were identified according to their waveform pattern and topography. This procedure improved the data quality somewhat but insufficiently, which was probably because movement artefacts are not stable enough over time, which is a prerequisite for computing the ICA components. Performing only the arm pull revealed for subject H a bilateral negativity; however, for subjects F and K, it revealed no interpretable results. For normal rowing, a negative activation at central sites appeared, indicating a motor potential, but at peripheral electrode sites, large activities occurred, which were only partly corrected using ICA. One possible source for artefacts in this study were electrode movements due to cable drag because cables were not fixed to the cap.

In summary, this pilot study revealed results similar to those of Study 1. Standard ERPs can be measured reliably during rowing; however, motor-related activity is largely distorted by remaining artefact sources.

## 4. Pilot Study 3

### 4.1. Introduction

Disadvantages of the two systems used in the previous pilot studies were the amount of required equipment and the data transmission via cable connections. Therefore, a study of ref. [33] was very promising. They used the Emotiv headset, a new developed wireless system (Emotiv, San Francisco, CA, USA, www.emotiv.com). After making some technical improvements, they measured ERPs in subjects walking around. The Emotiv system integrates the hardware in a small and lightweight headset, in combination with a wireless data transmission via a USB dongle to a laptop or even an Android smartphone [6]. Furthermore, this system integrates a two-axis gyroscope that measures head rotations. Electrodes and cables of the Emotiv system are fixed to stiff plastic arms, which should be effective to reduce artefacts by cable and electrode movements. Acceptable limitations of the Emotiv system, at least for pilot studies, are the fixed and lower sample rate and resolution and the lower number of channels (14). In addition, the sensor locations cannot be changed and are not well suited to sensorimotor research. Comparison studies revealed that the Emotiv system (equipped with the cheap original electrodes) performs less accurately than a medical device; however, it is able to record EEG data in a satisfying manner [34,35]. Comparing a modified (according to ref. [33]) Emotiv System with a commercial SynAmps System revealed only small differences, with a marginal worse performance of the modified Emotiv System [36].

### 4.2. Materials and Methods

**Figure 4:**
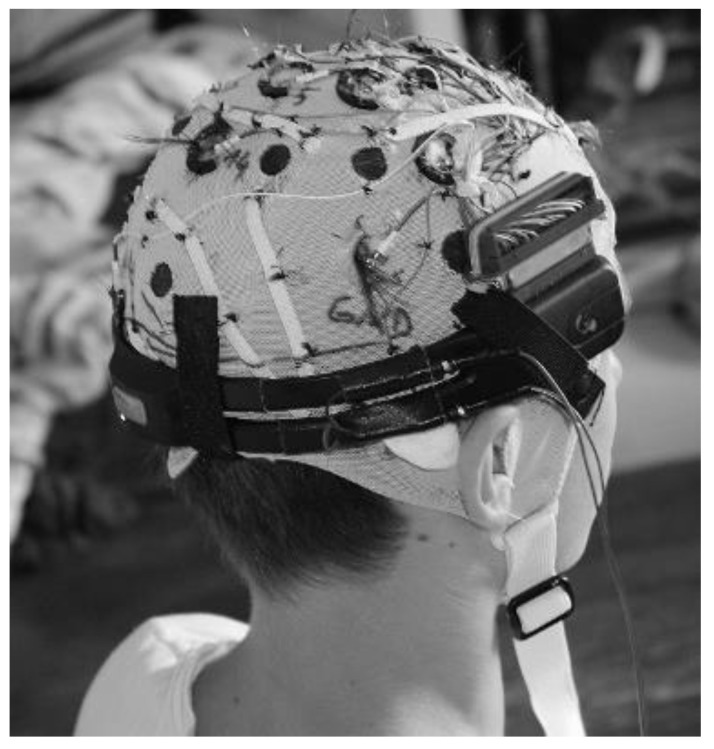
The modified Emotiv system combined with a standard electrode cap. A connector was fixed above the part of the headset containing the amplifier circuits. The two cables connected the headset with the photodiode used for the synchronisation of stimulation and EEG recording at the start and end of each experimental run. During rowing, the cables were removed.

As already practised by ref. [33], an Emotiv system was modified by removing the original sensors and plastic arms and connecting the system to the electrode cap with the gold electrodes used in Study 1 (Figure 4). A 14-channel EEG covering mainly the sensorimotor area (electrode sites AFz, Fz, FCz, Cz, CPz, Pz, FC1, FC2, CP1, CP2, C3, C4, O1 to assess VEPs, left mastoid, and reference right mastoid) was recorded using the Emotiv system (fixed settings: sample rate 128 Hz, resolution 14 bit/0.51 µV, bandpass filter 0.2–45 Hz, notch 50/60 Hz, digital 5th-order Sinc filter, data transmission 2.4 GHz band, and operation distance measured outside about 10 m). Signal quality was controlled with the Emotiv Testbench recording software. The ergometer, its measuring equipment, and the oddball task (except for a reduction of the number of standard stimuli from 180 to 120) were the same as in Study 1. The synchronisation of the EEG data with the biomechanical data and the visual stimulation was somewhat difficult. Although the Emotiv system can read trigger signals from a serial port, the laptops used did not have serial ports. An interface with serial-to-USB adaptors is not accurate enough in timing. Therefore, the Presentation scenarios were modified for synchronisation. A photodiode was fixed to the Presentation laptop and activated by stimuli at the beginning and the end of each experimental run. The triggers sent via the parallel port were recorded with a USB analog–digital device (RedLab 1208 LS, Meilhaus, Puchheim, Germany) together with the biomechanical data (force of the rowing stroke and movement of the sliding seat, sample rate 100 Hz). The signal of the photodiode (which was much larger than the EEG) was recorded with one EEG channel, and the cables were removed during the experimental runs. The EEG, biomechanical data, and triggers of the oddball stimulation were synchronised offline using purpose-written software. A separate channel for received data packets implemented in the Emotiv system provides the ability to control for lost data. This information was used to correct the synchronisation (a small fraction of samples was lost in 4 of 25 datasets). The EEG was analysed in a way similar to that used in Study 1 (using only a 20 Hz lowpass filter). Data of the rowing condition of two subjects had a larger number of trials contaminated with eye blinks. These were corrected using ICA without affecting other activities. The success of this procedure was controlled by comparing the EEG data from before and after the correction.

Four male subjects (aged 55, 21, 18, and 14 years, and referred to here as H, J, M, and D), with ergometer rowing skills allowing a high performance were instructed to perform six experimental conditions as follows: (i) oddball–rest; (ii) rowing with arm pull only (power 50 W, stroke rate (SR) 30/min); (iii) rowing 100 W/SR 20/min; (iv) oddball–rowing 100 W/SR 20/min; (v) rowing 180–200 W/SR 20/min; (vi) rowing 180–200 W/SR 26/min. Power and stroke rates could be controlled with the monitor of the ergometer and were close to the instruction. The rowing power ranged from recreational to long-distance endurance rowing. For subject D, the planned power thresholds were reduced to 60%. Written informed consent was obtained (respectively from a parent when under 18).

### 4.3. Results

**Figure 5:**
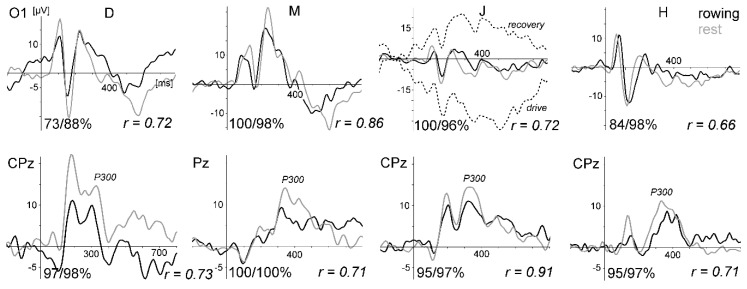
ERPs of the visual oddball task comparing rowing and rest for all four subjects (D, M, J, and H) of pilot Study 3. Upper graphs: VEPs evoked by the frequent checkerboard stimulus at electrode site O1 (referenced to Cz). Below: the P300 waveform at CPz/Pz (referenced to linked mastoids), which was larger during rest. The vertical line marks the stimulus onset. The numbers at the bottom of each graph present the percentage of artefact-free trials included in the averages for the rowing and rest of the chosen electrode site and the correlation coefficients. Deviant stimulus condition is not shown. For the frequent stimulus of subject J, the ERPs of the rowing condition, separated into drive (with force) and recovery phases of the rowing movement (without excluding artefactual trials), are presented additionally. In this recording, the VEP at electrode site O1 was overlaid by large movement-related drift artefacts, which cancelled each other out when all trials (drive and recovery) are included in the average waveform.

Figure 5 displays ERPs of all four subjects for the oddball task. As in studies 1 and 2, the VEPs were very similar between rowing and rest, the P300 was smaller during rowing, and the intraindividual differences were much smaller than the interindividual differences. The SNR values were always higher for rest than for rowing (VEP mean at O1: 0.159 vs. 0.054, t (3) = 13.5, *p* < 0.001). 

**Figure 6:**
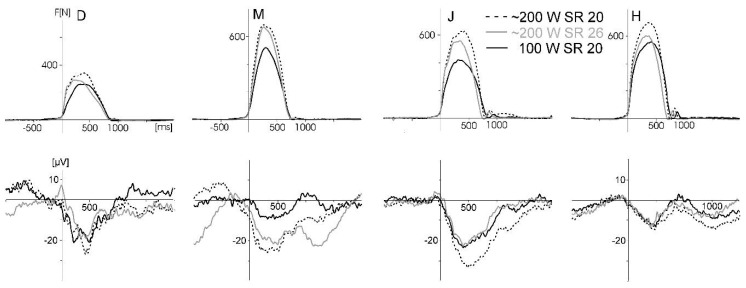
Force graphs (top row) and electrical activity (below) for the three different rowing conditions for all four subjects (D, M, J, and H) of pilot Study 3. Black solid: power 100 W, 20 strokes/min. Black dotted: 180–200 W, 20 strokes/min. Gray: 180–200 W, 26 strokes/min (lower power values for D). The electrical activity reflects the motor-related ERP (triggered by force onset, baseline −500 ms to 0) plus movement-related artefacts. Six bilateral sites (FC1, C3, CP1, FC2, C4, and CP2, referenced to the right mastoid) were averaged for this figure. The number of rowing cycles included in the different averages were between 54 and 88. Only a few EEG segments showing excessive and clearly identifiable artefacts (e.g., voltage jumps) were excluded.

The motor-related activity showed more or less large artefacts at several sites, as well as the expected modulation by rowing force at some other sites—that is, a larger negative activity with increasing force during the rowing stroke—with some differences between subjects (Figure 6). As it can be difficult to differentiate between motor-related activity and movement-related artefacts at sensorimotor sites, movement-related artefacts can clearly be identified at sites outside the sensorimotor region. Figure 5 displays such an example for subject J at electrode site O1. The VEP for the frequent stimulus of the oddball task was computed separately for the drive and recovery phases of the rowing movement (stimuli presented in the drive–recovery transition were excluded for this analysis). These waveforms were overlaid by large movement-related artefacts with reversed polarity, which cancelled each other out in the average, including all trials. These artefacts were independent of the chosen reference (Cz, right mastoid, and linked mastoids). That is, the overlay does not depend on physiological motor activity, which is much stronger at Cz than at the mastoids and O1; instead, it must be generated at electrode O1. Probably, this was due to cable artefacts in an electromagnetically noisy environment. Furthermore, the electrodes O1, left mastoid, and AFz had the largest distance to the Emotiv connector and therefore the longest cables, which may have allowed small movements.

**Figure 7:**
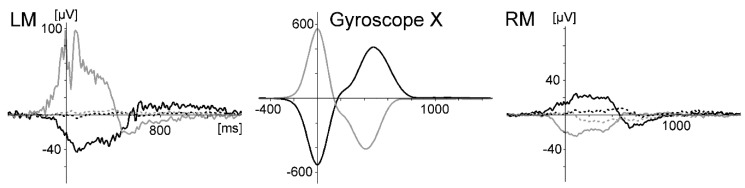
Movement artefacts generated by repeated rapid head rotations (measured with the gyroscope) at the left mastoid (LM, where the artefact was largest) and right mastoid (RM, both sites referenced to Cz). Rotations to the right side: black lines; rotations to the left side: grey lines. The polarity was inverted between left and right electrode sites (due to the Cz reference) and between left and right head rotations, which may depend on the activated part of the neck muscles (left or right). This artefact was present (with comparable amplitudes) at left sites C3 and O1, at right sites C4, FC2, CP2, and RM, and at O1, Fz, and AFz. Using independent component analysis (ICA)-based correction, the artefact could be removed nearly completely (dotted lines). The gyroscope was not calibrated (arbitrary units).

To identify one possible source of movement artefacts, the gyroscope of the Emotiv system was used to test if artefacts are caused by head movements. Therefore, rapid repeated movements were performed (left turn, right turn, and nodding) revealing very large artefacts, especially at lateral sites where the impact of the head movement was larger than at central sites. Using the Infomax ICA algorithm implemented in the Vision Analyzer 1.0 software, these artefacts could be strongly attenuated (Figure 7). A comparison of the gyroscope data for these head rotations and rowing showed only small head movements for nodding during rowing: that is, head rotations and the associated artefacts are not critical for rowing.

In summary, this pilot Study 3 revealed results similar to those of Studies 1 and 2. Standard ERPs can be measured reliably during rowing; however, motor-related activity is largely distorted by remaining artefact sources, whereas the use of, although modified, passive electrodes may be a limiting factor.

## 5. Pilot Study 4

### 5.1. Introduction

Under combining the technical advantages of Study 2 (active electrodes) and Study 3 (mobile headset), Study 4 followed two aims. (i) First, can movement artefacts be reduced when the passive electrodes of Study 3 are replaced by active electrodes, and can movement-related ERPs be measured in sufficient quality then? This would be the optimal result. (ii) If the first aim is not achieved, how is the signal quality of standard VEPs affected by movement dynamics (force output) and movement kinematics (movement speed)? That is, is there a trade-off between signal quality and movement intensity in the measurement of ERPs in e.g., cognitive tasks in moving subjects.

### 5.2. Materials and Methods

**Figure 8:**
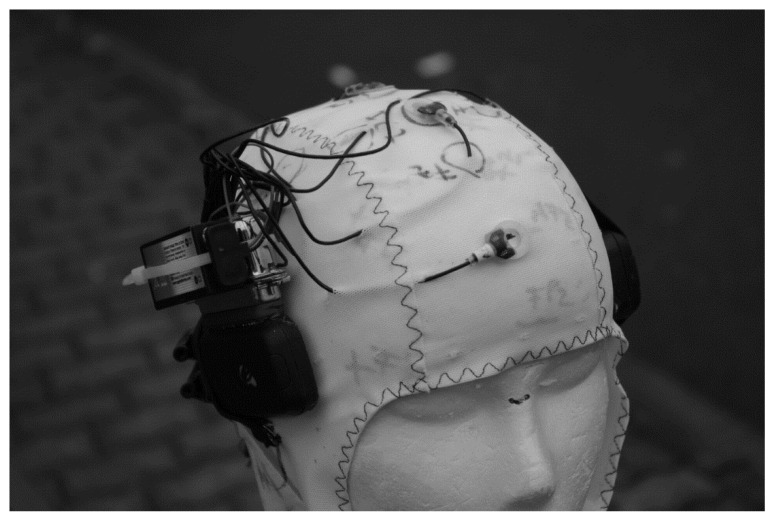
The modified Emotiv headset combined with the EasyCap active electrodes.

The Emotiv system used in Study 3 was combined with eight active electrodes (EasyCap active), which were provided by EasyCap GmbH (www.easycap.de) together with electrode caps (EasyCap). In contrast to the older version of the Acticap electrodes used in Study 2, these electrodes are smaller and very flat, and should therefore be less susceptible to tilting movements generated by inertial forces. After pretests, electrode cables were shortened, fixed to the cap, and connected to the headset. Power for the electrodes was provided by a 9 V battery attached to the connector (Figure 8). The electrodes were fixed with adaptors and a highly viscous conductive paste (Elefix). The active electrodes were placed at positions Cz, FCz, C4’, C3’, O1, left and right mastoid (TP9, TP10), focusing the measure of motor-related activity and the VEP. The active reference electrode was placed at AFz for different reasons: (i) it could be controlled by generating eye blinks if all electrodes worked properly; (ii) sections with eye blink and vertical eye movement artefacts could be detected and removed from the EEG data; (iii) a mastoid reference is largely affected by EMG artefacts generated by neck and head muscles; and (iv) the spatial distance to occipital sites is the largest and allows measuring larger VEPs. A disposable baby-ECG electrode, which served as ground/DRL (driven right leg), was placed at the right anterior temple. The original passive ground electrode of the EasyCap-active system was used as a signal electrode and placed at Oz. Of course, it is unusual to combine a passive electrode with an active reference electrode, and it was not certain whether it would work, because the amplifier would saturate if the impedances are very different. However, this electrode always provided a clear EEG signal, and no saturation effects were observed in any measure. Therefore, the quality of the VEP measured with the active electrode at O1 could be directly compared with the VEP measured with the passive electrode at Oz. Signal quality was controlled with the Emotiv Testbench recording software. The recording parameters (A-D rate 128 Hz, bandpass filter 0.2–45 Hz) of the ergometer, its measuring equipment, and the oddball task were the same as in Study 3 except for two modifications. The condition with the 60 deviant stimuli of the previous studies was removed because the VEPs did not differ from the standard checkerboard task. Therefore, the number of trials of the latter was increased from 120 to 180. Secondly, because no VEP signal was obtained from one subject, a second recording session was conducted at a later time (with a nearly identical rowing performance). To ensure that the visual stimulation was observed, the target stimulus (cross) was replaced by a lower number (27–33) of pictures of different airplanes. This was done because the oddball task was repeated six times instead of two times in the previous studies, and therefore, more salient stimuli were used. In this Study 4, the target condition of the oddball task was only used to control performance. 

**Figure 9:**
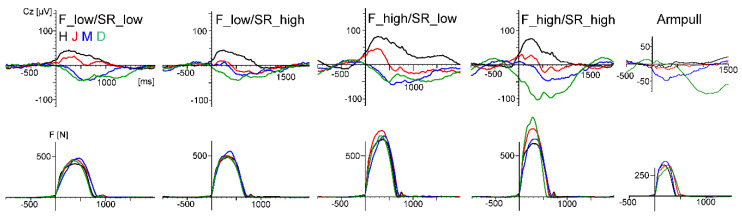
The upper row displays the force-onset triggered individual averages (subjects D, M, J, and H) of the ERP at Cz (referenced to AFz) and at the bottom, the averaged force graphs for the five rowing conditions (F: force, SR: stroke rate). ERPs at C3 and C4 were very similar to Cz and therefore omitted in this figure. The ERPs showed within-subject similarities with a higher activity in conditions three and four with the higher force output; however, there were large between-subject differences. The waveforms of subject D were largely affected by sweating artefacts in conditions four and six. Characteristics of rowing performance. Condition one: F_low, SR_low (individual mean values, range 19.4–21 min-1, 98–102 W). Condition two: F_low, SR_high (25.5–27.6), 121–163 W. Condition three: F_high, SR_low (19.8–21.3), 159–192 W. Condition four: F_high, SR_high (26.1–28.6), 225–270 W. Condition six: arm pull only (stroke rate 26.2–29.1), 50–57.5 W.

The same four male subjects as in Study 3 (aged 19, 23, 26, and 59 years, height 180–190 cm, weight 80–84 kg) performed six experimental conditions of about 4 min duration comparable to Study 3: (i) rowing with lower force output and lower stroke rate; (ii) lower force output, higher stroke rate; (ii) higher force output, lower stroke rate; (iv) higher force output, higher stroke rate; (v) visual stimulation in rest (without rowing); and (vi) rowing with arm pull only (details are provided in the legend of Figure 9). Power and stroke rates could be controlled with the performance monitor of the ergometer and were close to the instruction. The rowing power ranged from recreational (condition i) to long-distance (e.g., 10 km) racing (condition iv). The visual stimulation was applied in all six conditions. For comparison, one subject repeated all six conditions with the cap used in Study 3 with 14 passive electrodes.

Data processing was performed as in Study 3, using Vision Analyzer 2.1 (20 Hz lowpass filtering, segment lengths 1 s for VEPs, 3 s movement related, 2 s condition 6). VEPs were analysed at electrode sites O1 and Oz (referenced to Cz as in Study 3). The number of included trials (total 180) was on average 138 (O1) and 140 (Oz) (range 88–176). Movement-related activity was analysed relative to force onset and referenced to AFz. The number of rowing strokes was on average (across subjects and conditions) 104 (range 84–117). The number of included trials (sites C3, C4, Cz, FCz) was on average 63 (range 20–117). Excluded trials were mainly contaminated by ocular artefacts. Due to the low number of channels, an ICA-based artefact correction was not performed. The number of included trials in the movement-related condition was lower as in the VEP condition because of the longer segments (3 s vs. 1 s). One recording (subject D) was made on a hot August day. This resulted in large sweating artefacts, starting in condition three. Therefore, for the VEP analysis, the data of all recordings were filtered with a 1-Hz/24 dB highpass. This filtering did not visibly affect the morphology of the VEP waveform. For the analysis of the movement-related activity, no additional highpass filtering was made because at a stroke rate of 20/min movement, the frequency is about 0.33 Hz.

### 5.3. Results

**Figure 10:**
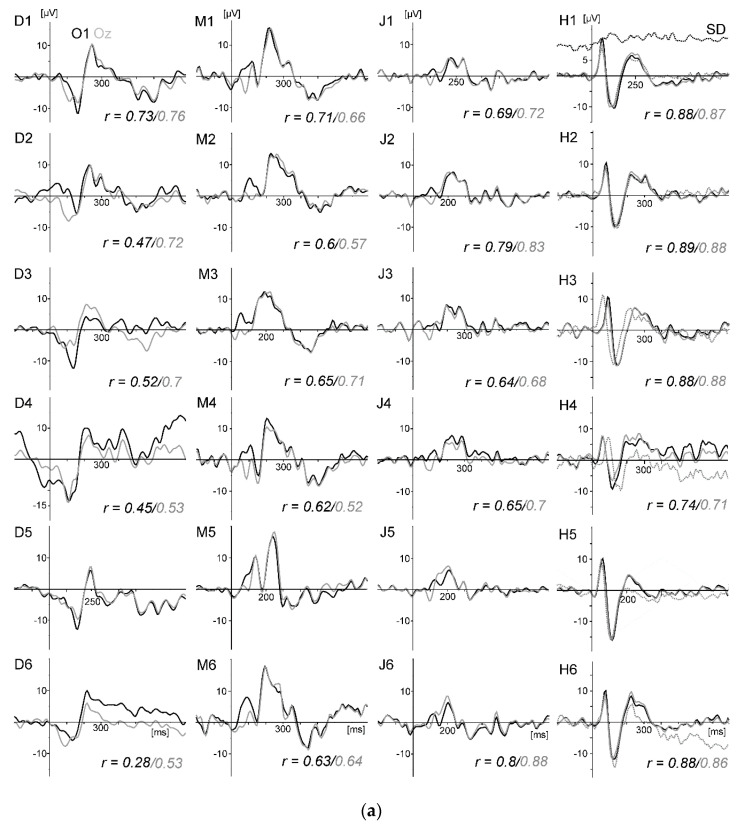

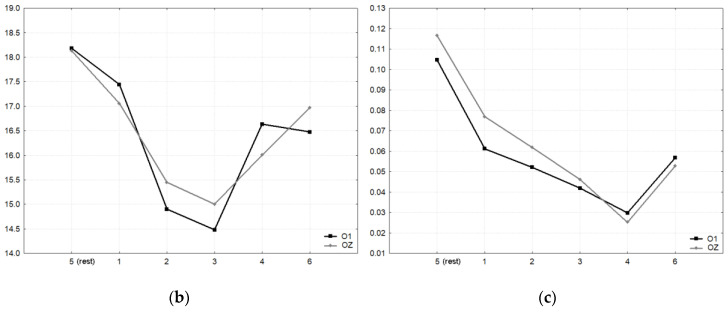
(**a**) VEPs generated by the standard stimulation (checkerboard) for all four subjects (columns, D, M, J, H) and the six rowing conditions (rows). The VEPs (referenced to Cz) measured at active electrode O1 and passive electrode Oz are superimposed. In addition, for subject H (right column), the VEP at O1 measured in the repeated test with the 14-channel cap with passive electrodes used in Study 3 is shown (dotted grey line). The six conditions were as follows: 1: Force low (F_low), stroke rate low (SR_low). 2: F_low, SR_high. 3: F_high, SR_low. 4: F_high, SR_high. 5: Rest (without rowing). 6: Arm pull only. Details are provided in the legend of Figure 9. The numbers at the bottom of the graphs present the correlation coefficients between the respective rowing condition and rest (after correcting latency delays up to 39 ms (mean 14.6 ms) between the two ERP waveforms, which sometimes occurred due to timing inaccuracies between the different hardware systems). (**b**) VEP peak differences (max–min) for conditions 1–6 (mean of all four subjects). The differences, which represent higher peak amplitudes, were largest in the resting condition (condition 5); however, the differences were not significant. (**c**) Signal-to-noise ratio (SNR) values for conditions 1–6 (mean of all four subjects). SNR decreased with increasing movement intensity. The rest condition differed significantly from all rowing conditions, as well as rowing conditions 1 and 4. No significant difference between both electrodes (O1 active, Oz passive) was found.

Figure 10a displays the VEPs generated by the standard stimulation (checkerboard) of the oddball task for all four subjects and conditions. For direct comparison, the VEPs measured at active electrode O1 and passive electrode Oz are superimposed. As in the previous studies, the VEPs were similar between rowing and rest, and the intraindividual differences were much smaller than the interindividual differences. Remarkably, the VEPs measured at O1 and Oz were highly congruent. Figure 10b displays the peak differences (maxima–minima in the interval 50 to 300 ms) of the VEP waveforms (mean of all subjects) for the six conditions. The SNR revealed the highest values in condition five (rest) and the lowest in condition four (Figure 10c). 

A statistical analysis was applied to these two parameters. For identifying within-differences between the six conditions and the two electrodes, we performed multilevel analyses with two within-factors conditions and electrodes and included random intercepts into the models. As a multiple comparison adjustment when performing pairwise comparisons of the conditions, Bonferroni was used. The results of the multilevel analyses were generated using SAS/STAT software, Version 9.4 of the SAS System.

For the peak differences, no statistical difference was found. For the SNR, the test of the main effect for each condition resulted in F (5,38) = 10.73, *p* < 0.0001; for the electrode effect, the test was not significant (F (1,38) = 0.60, *p* = 0.4415). After Bonferroni adjustment, the SNR differed significantly between condition five (rest) and all rowing conditions (*p* values between 0.032 and < 0.0001). Rowing condition four (highest power) differed additionally from condition one (*p* = 0.0236).

Figure 9 displays the motor-related activity at Cz. The waveforms show a systematic modulation by force output (and probably force-related artefacts) but not by movement speed (stroke rate). However, the large interindividual differences are physiologically less plausible.

The SNR data for these motor-related activities revealed no statistical difference. However, as indicated by the about ten times higher SNR values in comparison to the VEP waveform, the computation of the SNR could be misleading if an average waveform is dominated by a large and systematic artefact. Nevertheless, these data show that the quality of the ERP is lower when movement intensity increases, as is indicated in the VEP waveforms as well.

In summary, as shown in the previous studies, standard VEPs can be measured reliably during rowing, whereas signal quality decreases when movement intensity increases. The differences between the VEP measured at active electrode O1 and passive electrode Oz are marginal to small. This shows that active electrodes may not reduce movement artefacts further when electrode cables are shortened and fixated and a head-mounted amplifier is used. Consequently, the motor-related activity is still distorted by remaining artefacts.

## 6. General Discussion

The present pilot studies tested the practicability of four different EEG acquisition systems for ERP measures in moving subjects (a summary is given in Table 1). Technically, including the modifications, the used systems were suited to measure ERPs in moving subjects in contrast to conventional laboratory EEG hardware with passive electrodes. The advantages of the used systems were the head-mounted amplifier (studies 1, 3, and 4) and the active electrodes (studies 2 and 4), both methods reducing cable movement artefacts. However, as recently shown by ref. [37], increasing cable sway leads to a decrease in SNR, even when active electrodes are used; that is, to shorten and fix cables is essential. Meanwhile, a further advantage of available systems with low weight and small dimensions for movement research (research on moving subjects) is either wireless data transmission or storing the data on an SD card in the device itself. 

The data from fourteen single case measures from six different subjects revealed for a standard paradigm (visual oddball task) comparable intraindividual ERPs during rowing and during rest (non-movement condition) in all cases, despite remaining artefacts in the data. EEG parameters and ERP waveforms are genetically determined and show generally a broad range of interindividual differences, but they are also remarkably stable over time in adult subjects [28,38]. This fact (although probably not well known) strongly supports the reliability of the data, because the intraindividual differences were much smaller than the interindividual differences. Higher intraindividual differences instead would indicate that the ERP pattern is largely distorted by movement artefacts.

Artefacts were visibly larger in the raw data of the rowing condition(s) and quantified by considerably lower SNR values. The use of the SNR to quantify signal quality could be further extended. Computing the SNR from subsets of the data may inform about the minimum number of trials necessary for an experiment and may allow comparisons between different movement classes. Furthermore, because SNR is the relation between the power of the averaged ERP waveform and the power of the underlying EEG (see Appendix A), these two values may be helpful to identify if the ERP or the EEG is mainly affected by artefacts.

Whereas the VEPs were quite similar, the P300 was smaller during rowing than during rest (studies 1–3). This may be partly due to a habituation effect (the rest condition was always performed before the rowing condition), as recently suggested by ref. [39] as well, who used also an oddball task to compare ERP measures during indoor cycling with a resting condition. Another contribution to this effect was probably that multiple task demands reduce the P300 (e.g., as reviewed by ref. [40]). Here, attention is divided by counting the targets on the one hand and performing the rowing movement and the monitoring of stroke rate and power output on the other hand, which may be demanding for nonskilled rowers. Similar results with about a 30% smaller P300 amplitude during walking compared to sitting still (in counterbalanced order) were reported by ref. [33,41], who also suggested that the different task demands were the reason for this result. However, it has to be emphasised that the aim of the present pilot studies was not to investigate cognitive processes in rowing; instead, these robust ERP components (VEPs, P300) were measured for methodological reasons—that is, to compare ERP data quality during rowing and rest in a repeated measure design. 

The positive results of the oddball task, including the high intensive rowing conditions of study 4, are promising for the investigation of brain functions in naturally behaving subjects outside the laboratory: for example, in cognition research, brain–computer interface (BCI) applications, ambulatory assessment, and others. Since rowing is a very athletic sport and therefore a source of large movement-related artefacts, ERP measures with less motor activity such as walking around [33], walking or jogging slowly on a treadmill [15,18,19], or cycling when pedaling slowly at a subaerobic level [39] can easily be obtained using suitable equipment. Technically, non-movement related ERPs can be measured when active electrodes are combined with laboratory recording systems [3,4,15,18,19,21,39] or when passive electrodes are combined with head-mounted recording systems [5,22,33]. The reported analysis of movement-related activities in walking/slow jogging [19] or cycling were related to spectral analysis of the EEG, which is easier to assess than ERPs. Further related studies are reviewed by ref. [20].

Due to the nature of ecological settings, more limitations compared to laboratory settings have to be accepted. The placement of the stimulation monitor besides the ergometer and the oscillating viewing distance may be regarded as critical. Alternatives might be the use of a head-up display or an acoustical stimulation. However, the gyroscope data in studies 3 and 4 revealed only small head movements in the oddball task during rowing; therefore, it can be concluded that this was comparable in Study 1 and 2, because the placement of the monitor was the same. Eye movements were marginal or not present in the high-density recording of Study 2 but could be monitored at frontal sites in the other studies as well.

The (slight) differences in hardware settings, filter settings, and electrode positions were acceptable because reliability was assessed by the within-subject comparisons for each study separately.

The small sample size of these four pilot studies may be seen as critical from a cognitive neuroscience or psychological point of view. In those studies, mainly complex processes are investigated, which may differ between participants, will not be present in all participants, or may interfere with other factors. This will require a larger sample size and a statistical analysis to show if the found effects are random or if the probability is high that the effects are real. In contrast, the present studies used a salient visual stimulation with a full-screen reversed checkerboard. This stimulation reliably evoked a robust electrophysiological (sensory) response that cannot be suppressed when the stimulation is observed. The measure of VEPs is a valid test for the functioning of the visual system and a well-established tool for clinical diagnostics in neurology and ophthalmology [42]. Furthermore, a recent study proposed, based on ERPs measured in an oddball task, an ERP–EEG-based authentication system as an efficient biometric tool due to its convincing results [43]. In studies investigating higher cognitive processes (e.g., semantic processing) where an ERP component such as the N400, which is sensitive to these processes cannot be observed, researchers can use the VEP as a tool to check if the participants paid attention to the presented visual stimuli. This VEP, which was dominated by a negative and a positive peak in the interval between 100 and 170 ms, was obtained during rowing and rest in all fourteen measures, and it was therefore a valuable tool to compare signal quality between rowing and rest on an individual level.

The second and more challenging aim of the four studies was to test if motor-related activity could be measured. This approach is unique for these pilot studies, as the cited studies in the introduction aimed to measure cognitive processing in movement conditions, not the movement itself. Although motor potentials during the drive phase of the rowing movement, modulated by force output, were indicated (cf. Figure 6 and Figure 9), in all four studies, large movement-related artefacts occurred, which distorted motor-related activity. These artefacts can be identified at electrode sites apart from the pre- and primary motor cortex. In this context, it has to be considered that artefacts originating from the reference electrode will affect the other electrodes. As the classical mastoid reference captures EMG activity of head and neck muscles, reference electrode positions less affected by this EMG activity, as well as the activity of cortical motor areas, may be better suited (e.g., prefrontal sites or nose tip). Artefacts are more difficult to detect at sites covering the motor areas because muscle force generation, movement kinematics, and movement-related artefacts have the same time course. Therefore, further technical improvements to reduce artefacts beforehand or to identify artefacts better [44] and correct them are necessary to investigate motor behavior in movements including the whole body (as in sports: for example, motor learning or differentiating high from low performance in movement execution). One example to identify artefact sources was given in Study 3 when using the gyroscope to identify artefacts generated by rapid head movements. 

Known artefact sources are the EMG activity and sweating artefacts. The latter cannot be filtered out when movement frequency is in the same range (as seen in one subject of Study 4). Other sources of artefacts may rely on small movements of the electrode cables relative to the cap which were still possible; and the translational head movement during rowing in an electromagnetically noisy environment (the room was not shielded) may have generated small currents in the cables, as in a generator (according to Faraday’s law). However, in both cases, the active electrodes should be less vulnerable to this artefact sources. Furthermore, for the second case, artefacts should be larger when movement speed increases, which was not observed.

To investigate further possible sources of motion artefacts, ref. [45] used a phantom head to simulate motion artefacts in EEG data and found that artefacts increased with movement frequency as well as with movement amplitude, that is, in general with the acceleration of the phantom head. “We speculate that the major source of such artefacts is micro-movement of the recording electrodes in relation to the scalp surface“ [45]. Their data showed that artefacts strongly increased when the head acceleration was larger than 1.5 g. Based on these results, additional measures of head acceleration using a triaxial acceleration sensor (Move II, Movisens GmbH, Karlsruhe/Germany, www.movisens.com) attached to the Emotiv headset were analysed. These revealed values between 0.85 g in low-intensive rowing (75 W, 20 strokes/min) and 2.5 g in high-intensive rowing (360 W, 30 strokes/min). That is, the lower quality of VEPs in Study 4 at the rowing conditions with higher intensity may partly depend on such micro-movements of the electrodes independently of whether passive or active electrodes are used, because the main advantage of active electrodes is that these are less susceptible to cable sway artefacts.

Another pitfall with a physiological origin might be that different neuronal activities are superimposed, which hinders the detection of relevant activities. Ref. [23] observed that the amplitude of the motor potential correlates very high (r = 0.93) with force output in an isometric elbow flexion task and reported values of up to 8 µV/150 N. In rowing, these values (assuming a physiological source) are considerably higher (about 100 µV and 1000 N in Study 4). In contrast to these efferent activities, probably generated from the pyramidal cells in the primary motor cortex [46,47], other motor-related activities (originating from the premotor cortex or the SMA) are very small. For example, in two of our own visuomotor tracking studies, the effects related to motor learning were below 1 µV [12,13]. That is, if generally the activities related to motor learning or motor programming (e.g., in rowing the perception and adaptation of within-crew differences of rowing technique [27]) are in this amplitude range, these will be difficult to detect when the activity related to force execution is much higher. This hypothesis could be tested comparing rowing with other movement activities with a high force output, such as cycling on a stationary bike, which has the advantage that artefacts due to head movements and the related electrode movements could be minimised.

As a general recommendation for future ERP studies in an intensive-movement paradigm, cyclic movements where sub-movements (within one cycle) can be identified are advantageous. Movement kinematics and dynamics should be kept constant and can be controlled by providing feedback continuously about key performance factors (force, power, movement speed). Alternatively, after some training sessions to gain experience, participants should be able to control these factors intrinsically. For tasks (e.g., cognitive tests) not directly related to the movement itself, it has to be considered that movement intensity should be kept at the same level during the test. Furthermore, the test performance may decrease due to non-attention when the participant fatigues because intensity is too high.

## 7. Conclusions

The aim of the present set of four pilot studies was to examine if and to what extend of movement intensity ERPs could be measured in moving subjects. The two main questions were as follows. (i) Can ERPs not related to the movement be measured, i.e., to investigate cognitive processes in ecological settings? (ii) Can movement-related ERPs be measured to investigate neuronal processes e.g., associated with motor learning and motor control? Meanwhile, several other studies showed that ERPs related to cognitive processing can be measured when subjects are moving with lower intensity. The present study used a rowing task—a very athletic movement pattern with extensive head and body accelerations—that can be performed from a lower to a very high intensity. ERP signal quality (respectively the deterioration of the ERP) was assessed by comparing ERPs generated during resting and rowing. A salient visual checkerboard stimulation was used, which has the advantage of reliably evoking neuronal responses in the visual cortex (VEP) of healthy subjects. Additionally, in studies one to three, a P300 was measured which is easily detectable in most individuals. The results of all studies revealed that ERPs can be measured during rowing when the stimulation is not synchronised to the movement pattern, even when movement intensity (force and speed) was increased in study four, although the signal quality (SNR) decreased. This is a promising result for the investigation of cognitive or attentional processes (e.g., cycling in public traffic) in moving subjects. However, investigating higher cognitive functions (e.g., modulating N400 effects in language processing [48]) will require probably much higher sample sizes and an enlarged stimulus material than in typical laboratory settings. 

In contrast, the second aim was not reached—that is, to measure movement-related neuronal activity in sufficient quality. Although we tried to implement technical improvements based on the results of the preceding studies, signal quality remained poor, and further research is required. Comparing the equipment used in the four studies, a wireless headset combined with active electrodes is recommended because it reduces artefacts generated by cable movements and allows full mobility.

## Figures and Tables

**Table 1:** Overview of key indicators of the four pilot studies.

	Study 1	Study 2	Study 3	Study 4
**Amplifiers**	head-mounted	lab. standard	Emotiv headset	Emotiv headset
**Electrodes/Chans**	passive/20	active/61	passive/14	active/8 + 1 passive
**Number of Stimuli Frequent/Deviant/Target**	180/60/60	180/60/60	120/60/60	180/0/27–33
**Strokes/Min (Oddball Task)**	16–26	20–22	20–23	20–29
**Rowing Power (Oddball Task)**	moderate	moderate	moderate	moderate to <anaerobic threshold
**VEPs rowing/rest**	comparable	comparable	comparable	comparable
**MP/MRCP**	insufficient	insufficient	insufficient	insufficient

## Appendix A

Description of the computation of the SNR in the BrainVision Analyzer User Manual (Software Version 2.1.1):

The signal-to-noise ratio (SNR) is a measure of the quality of the EEG signal. Since there is no exact information available on either the signal or the noise in the EEG, their average total powers must be estimated statistically.

The average noise power of the EEG is first calculated for each channel. It is assumed that averaging eliminates the noise. For this reason, each EEG value in the original dataset is assigned the average value corresponding to its time position. Then, the square of the difference between the EEG value and the assigned average value is used as an estimated value for the noise power for each point.

The average noise power of each EEG channel is determined by calculating the sum of the estimated values for each point across all the data points of the channel in the original dataset and dividing the result by the number of points minus 1.

In order to ascertain the average power of the signal in the EEG, you first calculate the total power of a channel of the EEG. The total power is obtained by calculating the mean of the squares for all data points of the channel before averaging.

It can be assumed that the signal and noise are uncorrelated. Consequently, the average power of the signal is equal to the difference between the average total power and the average noise power.

The SNR is calculated from the quotient of the average signal power and the average noise power.
